# Benefits of influenza vaccine on stroke beyond preventing infection: Paradigm change or sheer bias?

**DOI:** 10.1111/ene.16239

**Published:** 2024-02-08

**Authors:** Mitchell S. V. Elkind, Francisco J. de Abajo

**Affiliations:** ^1^ Department of Neurology, Vagelos College of Physicians and Surgeons Columbia University New York New York USA; ^2^ Department of Epidemiology, Mailman School of Public Health Columbia University New York New York USA; ^3^ Clinical Pharmacology Unit University Hospital “Príncipe de Asturias” Madrid Spain; ^4^ Department of Biomedical Sciences (Pharmacology) University of Alcalá (IRYCIS) Madrid Spain

**Keywords:** epidemiology, influenza, stroke, vaccine

In 2006, the American Heart Association and American College of Cardiology jointly recommended annual vaccination against influenza for patients with coronary artery disease to prevent recurrent cardiovascular events [1]. Those recommendations were based on a single randomized clinical trial [2] and observational studies. Subsequent observational studies, trials, and meta‐analyses—while not unanimous—have provided further supportive evidence that influenza vaccination prevents recurrent events in patients with coronary artery disease. In a meta‐analysis of eight trials (*n* = 14,420 patients), influenza vaccination, compared with standard care, led to a 25% lower risk of major adverse cardiovascular events [3]. This risk reduction is comparable to that of other standard preventive therapies, such as statins and smoking cessation [3]. By 2020, the European Society of Cardiology also endorsed influenza vaccination to prevent recurrent cardiovascular events in patients with coronary artery disease [4]. Although these recommendations applied to patients with cardiovascular disease, clinical practice guidelines have remained relatively silent about the potential role of influenza vaccination to prevent recurrent stroke. The 2014 American Stroke Association (ASA) guidelines for the primary prevention of stroke, for example, give only a Class IIa recommendation for influenza vaccination [5], and the 2021 ASA secondary stroke prevention guidelines do not mention influenza vaccination at all [6].

A growing body of literature provides evidence, nonetheless, that seasonal influenza may transiently increase the risk of ischemic stroke. [7, 8] Thromboinflammation, a procoagulant state driven by inflammatory response to influenza and other viruses, is a likely mechanism for this increased risk [9]. Endothelial dysfunction and atherosclerotic plaque instability may also play a role. If influenza can trigger stroke, however, then vaccination against influenza would be expected to reduce stroke risk. Indeed, a recent meta‐analysis of 26 trials and observational studies found that influenza vaccination was associated with an approximate 20% reduction in risk [10], though further studies, including randomized trials, testing efficacy of vaccination for stroke prevention are warranted.

One concern of influenza vaccination, however, is that vaccination may itself transiently increase inflammation, potentially increasing short‐term stroke risk, even as it reduces long‐term risk. Vaccination, in other words, could serve as a stroke trigger despite long‐term benefits. A study by Tanaka and colleagues in this issue of the *European Journal of Neurology*, however, lays this concern to rest [11]. The investigators conducted a large‐scale administrative database study in Alberta, Canada, using health insurance data on all adults in the province, covering the period from September 2009 to December 2018. They explored the risk of stroke during time windows of 3, 7, 14, 21, and 30 days after recorded vaccination against influenza, and analyses were adjusted for demographics and common stroke risk factors. The population at risk was large: among 4,141,209 adults, approximately 43% (nearly 1.8 million persons) received at least one annual vaccination. There were 38,126 strokes, of which 1309 occurred within 30 days of vaccination. The risk of stroke was significantly reduced after vaccination for each time window, including 3 days. The authors concluded that influenza vaccination reduces stroke risk at all timepoints.

The results are reassuring that influenza vaccination is unlikely to cause short‐term increased risk of stroke. Strengths of the analysis are the large number of people in the source database and the availability of data on vaccination and several demographic and clinical risk factors. There are limitations, as well, however. While the overall number of vaccinated individuals was large, the numbers of those with stroke and vaccination within each time window, which drives the associations, are smaller. Time windows explored, moreover, were not mutually exclusive: the window of 7 days, for example, overlaps the 3‐day window, impeding a precise assessment of temporal effects. Also, the potential confounders considered excluded smoking, body mass index, and use of antiplatelets or statins, leaving room for residual confounding.

Another interesting outcome of the results is that potential benefit on stroke prevention occurred well before an immune response against infection takes place, usually established as 10–14 days, excluding the possibility that the early reduced risk observed can be explained by prevention of influenza infection itself and pointing, perhaps, to a direct effect of vaccination on the pathophysiology of acute atherothrombosis. Others [8] have also observed similar early effects of influenza vaccination against stroke and myocardial infarction in a self‐controlled case series study, a design that adjusts for factors that do not rapidly change over time. A possible direct effect of influenza vaccination was also suggested by Rodríguez‐Martín et al. [12], in a nested case–control study utilizing a nationally representative primary care database from Spain that analyzed the relationship of influenza vaccination and ischemic stroke during pre‐epidemic, epidemic, and post‐epidemic periods over 15 influenza seasons (2002–2015). They observed a moderate protective effect of influenza vaccine on incident ischemic stroke after adjusting for potential confounders during the epidemic periods (as expected), but also during pre‐ and post‐epidemic periods, providing indirect evidence that the mechanism of benefit might be a more general immune‐related effect rather than one specific to influenza itself.

All these studies, however, are observational in nature, and bias can be a plausible explanation of the “benefits” of influenza vaccination. In real‐life conditions, those who undergo vaccination may be healthier or have healthier habits than those who do not. Also, fragile patients and those at higher risk of atherothrombotic events might defer vaccination because of other illness. These phenomena may create a scenario whereby vaccinated patients are at baseline lower risk of suffering stroke than unvaccinated patients, leading to apparent protective effects. Future studies should elucidate whether influenza vaccination has a direct effect beyond prevention of infection itself. The possibility of a randomized clinical trial in patients at high risk of stroke, similar to those in coronary disease [2], should be considered.

The results from Alberta, meanwhile, provide further evidence that vaccination rates are low overall. Only about 43% of people in the region received the vaccine, a proportion similar to that in Spain. Recent concerns, including disinformation efforts about adverse vaccine effects since the coronavirus pandemic, could further reduce these numbers. These data could therefore be used to further encourage clinicians to recommend influenza vaccination to high‐risk populations; the potential benefits of influenza vaccination on stroke risk—and the absence of a short‐term risk—only make that message more urgent.

## AUTHOR CONTRIBUTIONS

**Mitchell S. V. Elkind:** Conceptualization; writing – original draft; writing – review and editing; investigation. **Francisco J. de Abajo:** Writing – review and editing; investigation; conceptualization.

## CONFLICT OF INTEREST STATEMENT

F.J.A. reports receiving an unrestricted research grant from Sanofi‐Pasteur for work on influenza and myocardial infarction. M.S.V.E. has no disclosures.

## Data Availability

Data sharing is not applicable to this article as no new data were created or analyzed in this study.

## References

[1] Davis MM , Taubert K , Benin AL , et al. Influenza vaccination as secondary prevention for cardiovascular disease: a science advisory from the American Heart Association/American College of Cardiology. Circulation. 2006;114:1549‐1553. 16982936 10.1161/CIRCULATIONAHA.106.178242

[2] Gurfinkel EP , Leon de la Fuente R , Mendiz O , Mautner B . Flu vaccination in acute coronary syndromes and planned percutaneous coronary interventions (FLUVACS) study. Eur Heart J. 2004;25:25‐31. 14683739 10.1016/j.ehj.2003.10.018

[3] Yedlapati SH , Mendu A , Tummala VR , Maganti SS , Nasir K , Khan SU . Vaccines and cardiovascular outcomes: lessons learned from influenza epidemics. Eur Heart J Suppl. 2023;25(Suppl. A):A17‐A24. 36937374 10.1093/eurheartjsupp/suac110 PMC10021491

[4] Knuuti J , Wijns W , Saraste A , et al. 2019 ESC Guidelines for the diagnosis and management of chronic coronary syndromes. Eur Heart J. 2020;41:407‐477. 31504439 10.1093/eurheartj/ehz425

[5] Meschia JF , Bushnell C , Boden‐Albala B , et al. Guidelines for the primary prevention of stroke: a statement for healthcare professionals from the American Heart Association/American Stroke Association. Stroke. 2014;45:3754‐3832. 25355838 10.1161/STR.0000000000000046 PMC5020564

[6] Kleindorfer DO , Towfighi A , Chaturvedi S , et al. 2021 Guideline for the prevention of stroke in patients with stroke and transient ischemic attack: a guideline from the American Heart Association/American Stroke Association. Stroke. 2021;52:e364‐e467. 34024117 10.1161/STR.0000000000000375

[7] Smeeth L , Thomas SL , Hall AJ , Hubbard R , Farrington P , Vallance P . Risk of myocardial infarction and stroke after acute infection or vaccination. N Engl J Med. 2004;351:2611‐2618. 15602021 10.1056/NEJMoa041747

[8] Boehme AK , Luna J , Kulick ER , Kamel H , Elkind MSV . Influenza‐like illness as a trigger for ischemic stroke. Ann Clin Transl Neurol. 2018;5:456‐463. 29687022 10.1002/acn3.545 PMC5899905

[9] South K , McCulloch L , McColl BW , Elkind MS , Allan SM , Smith CJ . Preceding infection and risk of stroke: an old concept revived by the COVID‐19 pandemic. Int J Stroke. 2020;15:722‐732. 32618498 10.1177/1747493020943815 PMC7534199

[10] Zahhar JA , Salamatullah HK , Almutairi MB , et al. Influenza vaccine effect on risk of stroke occurrence: a systematic review and meta‐analysis. Front Neurol. 2024;14:1324677. 38269000 10.3389/fneur.2023.1324677 PMC10806129

[11] Tanaka K , Demchuk A , Malo S , Hill MD , Holodinsky JK . Risk of stroke within 3, 7, 14, 21 and 30 days after influenza vaccination in Alberta, Canada: a population‐based study. Eur J Neurol. 2024. 10.1111/ene.16172 PMC11236019 38117538

[12] Rodríguez‐Martín S , Barreira‐Hernández D , Gil M , García‐Lledó A , Izquierdo‐Esteban L , De Abajo F . Influenza vaccination and risk of ischemic stroke: a population‐based case‐control study. Neurology. 2022;99:e2149‐e2160. 36240087 10.1212/WNL.0000000000201123

